# Developing a Real-Time Working Method That Improves Process Efficiency in High-Power Fiber Laser Systems

**DOI:** 10.3390/mi13091552

**Published:** 2022-09-19

**Authors:** Uğur Yalçın, Uğur Karanfil

**Affiliations:** 1 Electrical-Electronic Engineering Department, Bursa Uludag University, Bursa 16059, Turkey; 2 Department of R&D, Durmazlar, Electrical-Electronic Engineering Department, Bursa Uludag University, Bursa 16059, Turkey

**Keywords:** materials processing, fiber lasers, laser cutting, laser piercing, cutting parameter, photodiode, monitoring

## Abstract

The need for studies on new simulation and monitoring methods for interactions occurring during material processing in high-power fiber laser systems has increased. In this manuscript, a structure that can intervene in real time and improved solutions that demonstrate the potential of photodiode-based monitoring are presented. By processing the signals instantly received during material processing with InGaAs and Si photodiodes integrated into the cutting head in algorithms, the method that intervenes in the process by detecting the last stage of the piercing process and the problems that may occur during cutting are explained. The stability of the proposed system has been tested on the most used materials in the industry such as St37, stainless steel, and aluminum at laser powers of 6, 8, and 10 kW, respectively. In this article, it is shown that there is a relationship between the signals observed in the infrared (IR) and visible (VIS) spectrum and the characteristics of the cut quality and scenario. Analysis results of photo-diode tracking signals obtained according to material, power, and gas type are presented. Among the innovations added by the method are related application process improvements, material analysis, and cutting and piercing parameter improvements.

## 1. Introduction

The low costs of non-contact processing, high cutting speed and accuracy, and low energy requirements have encouraged the widespread use of lasers in metal processing [1,2,3]. Laser applications are used in many different processes such as cutting [4,5], welding [6], coating [7], drilling [8], and marking [9]. With the advantages of Computer Numerical Control (CNC), 2D or 3D parts and pipe or profile processing applications have become widespread. Aside from industrial metal material processing, the scope of application for laser and optical technologies is also quite wide, including optical communication and lithography, medicine, aesthetic medicine, the military, and even food packaging [10,11].

Industrial applications of laser and optical technologies have grown significantly in recent years. Although high-power fiber laser technology emerged at the beginning of the twenty-first century, it soon became a focus of attention. According to statistics provided by Laser Focus World, laser systems for materials processing reached a record volume in 2021: USD 21 billion. Looking at the market segments, high-power laser cutting and welding systems are considered to have by far the largest share in almost half of the market [12].

The laser cutting process is a material processing technique used in academia as well as industry. Line or burr defects may occur as a result of the process, which can reduce the final quality of the part being cut. Thus far, many studies can be found in the literature on the subject. The first steps toward the cut monitoring method were taken by Jorgensen in 1991, who presented cut analysis based on light emitted from the kerf [13]. Sforza later published a similar study on infrared [14]. In the following years, first and second law efficiencies related to kerf were suggested [15]. Thermal and analytical models [16,17] were also used to characterize the material processing phenomenon, and the laser parameters and melting direction were investigated [18].

Since light matter interactions that occur during the process are non-linear and difficult to model mathematically, new simulation and monitoring methods need to be developed [19]. In relation to this, a method that takes into account heat transfer with IR cameras has been proposed [3]. In addition, acoustic optical signals and high-speed imaging techniques are among the preferred methods in current research [20,21,22]. On the other hand, previous independent studies in the literature have presented outputs showing that the use of photodiodes works with high detection, sensitivity, and stability [23,24,25,26]. These sensors are reliable devices that can be used as piercing detection and cut quality indicators in laser material processing applications [27].

In high-power fiber laser applications, it is an inevitable need to add real-time software solutions and additional sensors to enable more advanced functionalities. This affects not only process improvement and efficiency, but also the lifetime of components. In addition to having a low cost and easy installation, photodiodes can detect process emissions over a wide wavelength range. High error detection rates with minimal error make photodiodes a desirable choice for monitoring high speed laser processes [27].

Different process parameters are used for each material type and thickness in laser cutting systems. Laser power, duty, frequency, stand-off distance, nozzle diameter and type, and gas pressure and type are the main parameters. However, apart from these, the processing time (waiting time) in the piercing process required to start cutting is determined as fixed. A certain factor of safety (typically up to 3×) is added to the average wait time. While not necessary for most piercing processes, it is vital for piercing stability in conventional systems where there is no structure to detect the end of the process. In the cutting process after piercing, there is no solution for the negative consequences (deterioration or overheating of the material surface, decrease in laser power, etc.) that may occur in the absence of a system that monitors the process and intervenes instantly.

In this study, photodiode monitoring signals were analyzed by varying laser power, material type, and process gas. Studies have been carried out to increase knowledge of material processing with high-power lasers, and a method that can instantly intervene in the piercing and cutting process is presented. Since the signal values used as a reference in the cutting process are obtained from quality cuts, there is no hesitation about the preservation of quality.

The previous study focused on low power, but for high powers such as 8 and 10 kW, it was necessary to improve the algorithms, increase the functionality, filter the signals, and increase the sampling rate. In this study, the stability of the method was tested with three different laser powers and many different materials. The working logic of the piercing detection system is explained in detail in the graph.

## 2. Materials and Methods

A Durma HD-F 3015 flatbed cutting machine with Precitec ProCutter cutting head was used as the machining workstation in the tests. The 6, 8, and 10 kW IPG YLS CUT series were used to generate laser power, respectively, and laser cuts were made with 100 micron fiber. The technical specifications and optical characteristics of the devices used are shown in Table 1 and Table 2. Although the data provided by the manufacturer are shared in the tables, it should be known that in the measurement process, the measured values cannot represent the actual values. The results obtained also include some possible errors. These mistakes will not happen all at once, and the weight of their effects on the result cannot be at the same level. The uncertainty of measurement defines the range in which the measured values are around the mean value with a certain probability. While uncertainty is usually given as a figure, it can be absolute or relative [28,29]. The focus lens in the high-pressure automatic cutting head is protected from particles generated during the cutting process by using a low-cost protective glass.

The size of the sheet metal was ignored, but in all tests, a workpiece with the same geometry and dimensions was cut. Pure (99.99%) oxygen gas and nitrogen gas were used as auxiliary gases for piercing and cutting operations. Due to the step piercing strategy, focus, pressure, and nozzle distance were changed according to the steps. Thanks to this strategy, these variables can be applied to the material in different combinations for the desired period of time. Generally, long period and high nozzle distance are preferred in the first steps, while close distance and cutting focus are preferred for cutting preparation in the last steps. In this way, sheet heating, slag formation, and optical contamination are prevented, and the process is continued with ready-to-cut parameters. The laser beam was carried by a standard fiber optic cable. The polarization state may simply be unstable, for example due to temperature shifts, or it may switch randomly between different directions. However, it was not considered for this study, as it would not have a major impact on the study. The maximum cutting speed was set according to the condition that the workpiece to be cut can be completely separated from the entire plate.

**Figure 1:**
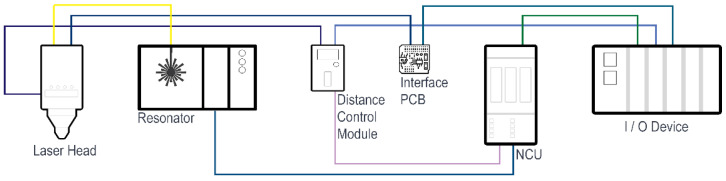
Schematic diagram of equipment used for tests.

Edge cut and piercing quality were determined by visual inspection, and the surface qualities of the test samples are presented in Appendix A. In order to observe the process, InGaAs (1000–1800 nm) and Si (400–1100 nm) photodiodes integrated into the cutting head and measuring the light intensity reflected back from the process surface were used. The technical specifications of the cutting head are given in Table 3. Figure 1 shows the schematic diagram of the study, where the yellow line represents the fiber cable. Other wiring consists of digital and analog signals. Distance control module and PCB Interface are components of the cutting head. In the study, Siemens 730.3 was chosen as the NCU. ET200 SP series and high-speed digital and analog modules are used in the I/O group.

## 3. Real Time Monitoring System and Algorithm Structure

The working principle of the system is generally based on the sensors monitoring the back reflection of the laser power on the material surface during piercing and cutting. The sensor outputs are processed in the NC and PLC software created on the CNC machine, and the process is instantly controlled as a closed loop.

**Figure 2:**
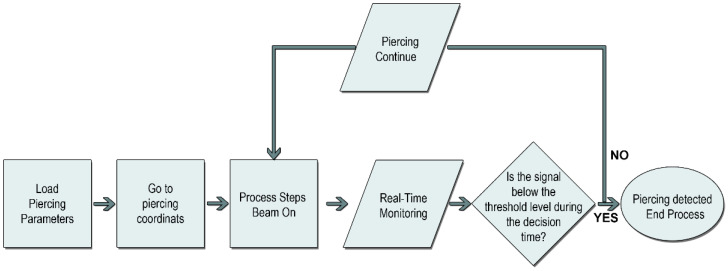
Piercing detection algorithm in high-power fiber laser systems.

In the piercing process (Figure 2), the laser parameters to be used in the material are first loaded into the machine. These data include many parameters such as focal length, cutting gas and pressure, nozzle distance, and laser power. The machine manages these values in the background during the process through the numerical controller and PLC. The threshold values determined by measuring are also uploaded to the tracking system at this stage. Instant monitoring system is activated when the laser is turned on. The threshold values are constantly compared with the instantaneous feedback values received from the sensors. If the instantaneous measured value remains below the threshold value for the defined time, the system detects the completion of piercing and generates a signal in response. The CNC machine turns off the laser beam, completes the process, and sets the necessary parameters for cutting. The laser beam is controlled by the fast input and output modules on the numerical control unit (NCU) of the CNC machine. The NCU’s input process output (IPO) cycle time is 1 ms. In the resonator, the laser beam control is in the order of microseconds, so the beam closing time is provided under 2 ms.

**Figure 3:**
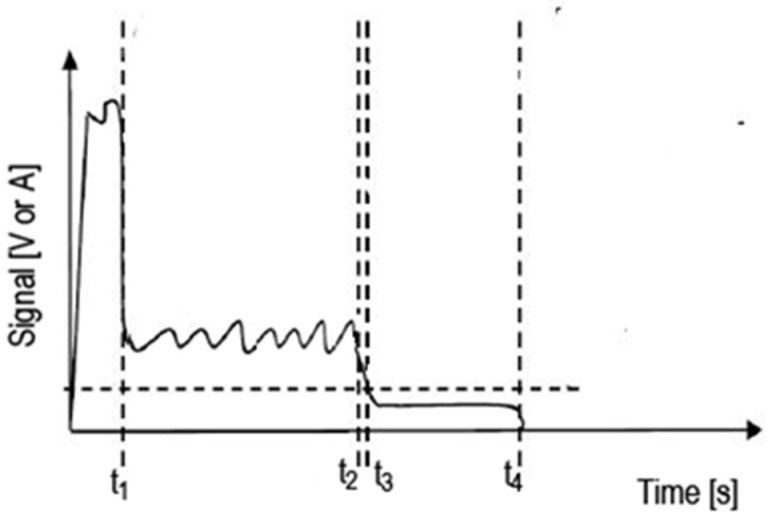
Typical signal recorded by photodiodes during the piercing process.

Figure 3 shows a typical example of a recorded signal in the piercing process. With the first light coming out, a high amplitude signal is measured at time t_1_. This is due to the laser hitting the surface for the first time. Between the times t_1_ and t_2_ the material has started to gouge, and unstable back reflection signals are measured depending on the cavity shape and the position of the cutting head. The moment t_3_ indicates that the laser beam has started to pass to the back surface of the material for the first time. From the moment t_3_ to the moment t_4_ when the laser is turned off, low-level back reflection signals are observed, even if they are not zero. The piercing detection method works depending on the determined threshold signal level (t_3_ moment) and programmable decision time. Decision time is just as important as the threshold level. In cases where the threshold level is not selected correctly, the value read may rise above this level momentarily after falling below this level. To determine this level, the signals of the standard piercing process are recorded with the system proposed in the study. The signal level of the t3 moment is determined in the recorded graphics and the stable decision state between t3 and t4 is sought. This interval actually represents the wasted time when drilling is complete. The decision time, on the other hand, is determined according to the difference in the time between which the slope of the decrease of the level at the time t3 ends and the stable signal movement begins to be observed.

**Figure 4:**
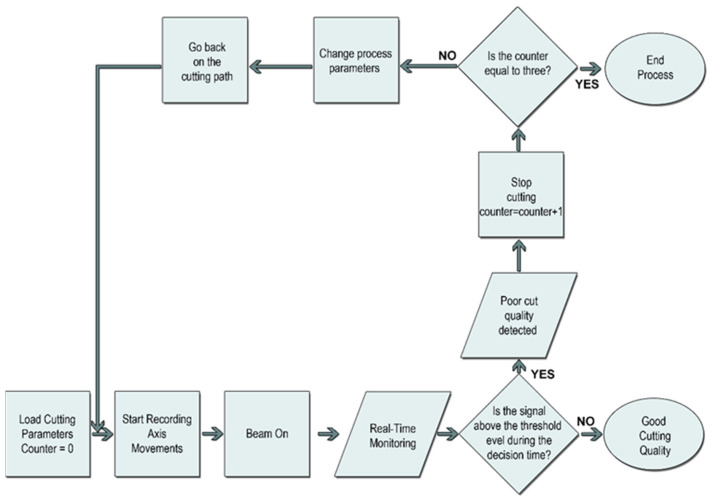
Cutting monitoring algorithm in high-power fiber laser systems.

In the method (Figure 4) that instantly monitors the cutting, unlike the piercing process, it is possible to stop the process, rewind, and automatically change the applied parameters. In the related structure, first, the cutting parameters are loaded on the machine and the bad cut detection counter value is set to zero. With the recording of the axis movements in the machine, the values from the sensors are also read when the laser beam is turned on. If the signals read until the end of the cut remain above the threshold level for more than the decision time, the system interprets this as a poor-quality cut. In this case, by increasing the counter value, a certain amount is retraced on the workpiece path, cutting parameters are changed (cutting speed is reduced by 15% or gas pressure is changed), and cutting is tried again. If a weak cut is detected despite two interventions, the process is stopped. This stops the cutting or skips to the next part or contour according to the predefined scenario. Meanwhile, subprograms written in the numerical controller allow the original cutting parameter values to be loaded while switching between parts and contours. If the same problem is encountered in the other part it passes through, the system stops the machine completely and warns the operator with an alarm message.

## 4. Measurements and Discussion

In order to ensure the accuracy of the proposed smart algorithm software, many materials were cut in different thicknesses and strengths, measurements were taken, and a database was created by saving these measurements to the system. In addition to step piercing, the imported records were created from straight, curved, and angular cuts. The laser beam was carried by a standard fiber optic cable, and the maximum cutting speed was set under the condition that the workpiece to be cut can be completely separated from the entire plate. Edge cut and piercing quality were determined by visual inspection. During the tests, the cutting process parameters, such as the height of the cutting head to the sheet metal, the cutting speed, the position of the focus, the laser power, the gas pressure and the nozzle type, were changed to determine the direct relationship between the parameter values and the response of the monitoring signal; different values were tested, maintaining a good cut quality. The tests revealed that there is no single parameter that provides a good quality cut, but that flexibility can be achieved with certain combinations.

Firstly, tests were carried out on stainless steel and aluminum to observe the instantaneous effect of the material on photodiodes with 6 kW laser power. The sample parameter set used is given in Table 4.

**Figure 5:**
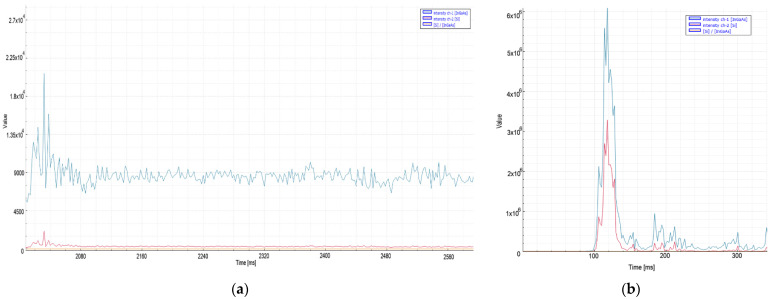
Measurement values recorded during nitrogen cutting of 8 mm aluminum material: (**a**) quality cut with ideal parameters, (**b**) the moment of deterioration of cut and quality.

It was observed that (Figure 5) when factors such as the type of auxiliary gas pressure, material thickness, and laser power were not changed, stainless steel exhibited a higher tracking signal value than aluminum in both spectra. This is related to the higher emission coefficient of the material and is compatible with the literature [30].

In the case of a deterioration in quality in 8 mm aluminum material, high peak levels were recorded instantaneously at the signal level. The fact that both of them are cut with nitrogen increases the acceleration of the signal level read by the sensors in the case of deterioration. In aluminum material, 1 × 10^6^ and 25 ms were chosen for threshold level and decision time, respectively. When choosing these values, the instantaneous rises at the moment of the quality cut, and the peak levels and peak widths of the signal at the moment of the bad cut, are important.

**Figure 6:**
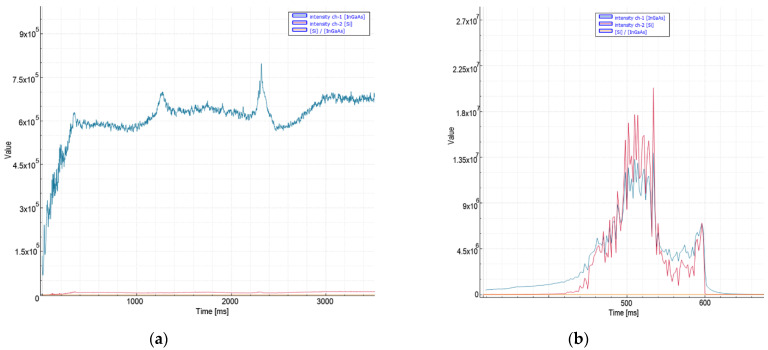
Measurement values recorded during nitrogen cutting of 8 mm stainless steel material: (**a**) quality cut with ideal parameters, (**b**) the moment of deterioration of cut and quality.

In cases of a deterioration in quality in 8 mm stainless steel cuts, very high peak levels were instantaneously observed (Figure 6), especially in the VIS spectrum (1.8 × 10^7^). A threshold level 1 × 10^7^ is selected for bad sector detection and 20 ms for decision time. Thanks to the method recommended in 8 mm aluminum and stainless steel materials, which were traditionally pierced in about 1 s, an average improvement of 0.25 s was achieved in the process.

**Figure 7:**
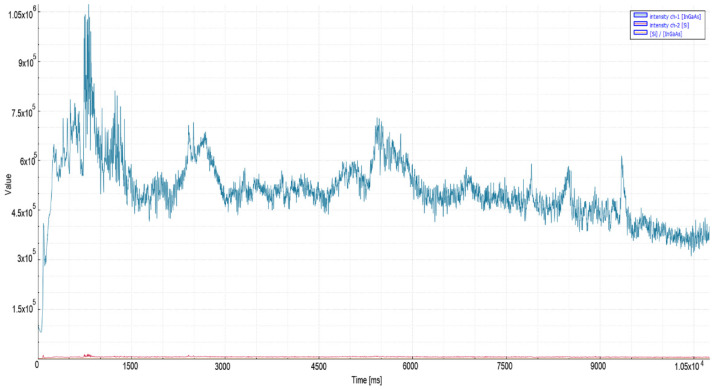
Measurement values recorded during oxygen cutting of 20 mm mild steel material.

**Figure 8:**
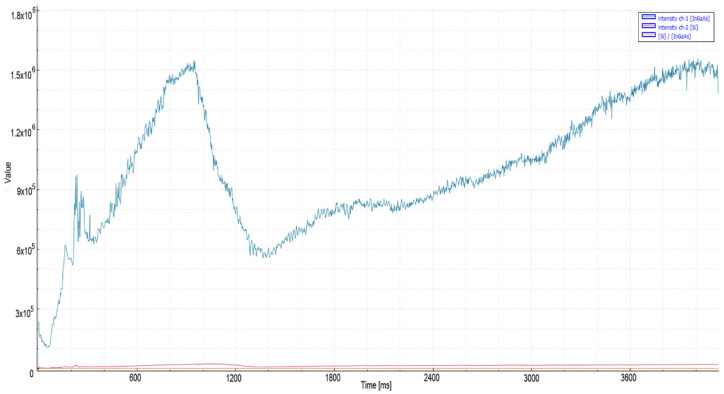
Poor quality oxygen cutting measurement values of 20 mm mild steel material.

In order to examine the effects of increasing laser power when applied to the same material, 20 mm mild steel material was cut with 6 kW laser power. As seen in Figure 7, a stable signal amplitude of the order of 6 × 10^5^ was observed in the IR spectrum during quality cutting after the piercing process. In poor quality cutting, on the other hand, with the increase in material temperature after piercing, a gradual increase was observed in the signal level, and the peak values reached 1.5 × 10^6^. According to the results obtained, a threshold level of 8 × 10^5^ and 25 ms stability time was found to be suitable for the relevant material. This recording, shown in Figure 8, exhibited the same characteristics as the 20 mm ST37 tests in the previous 4 kW study [2].

In mild steel, overheating the sheet surface leads to distortion of the pierce zone and enlargement of the hole diameter. It is important to set the process parameters and to perform the piercing in a step structure, especially for thick materials. While analyzing the results, traditional parameters were not deviated, and all piercing tests were successfully completed.

In the next stage, cuts were made on 15 and 25 mm mild steel materials with 8 kW laser power. The parameter sets used are given in Table 5. Mild steel is a challenging material for both the piercing and cut monitoring process due to the reaction of oxygen. Tests were conducted on a very thick material, such as 25 mm, to increase this level of difficulty.

In 25 mm mild steel material, which is traditionally pierced with four steps, the piercing process is detected at the beginning of the fourth step thanks to the proposed method, and an average speed of 0.5 s per piercing is achieved. In 15 mm mild steel, an average of 0.3 s per hole was accelerated in three steps. Assuming that the process will contain 3 × 1.5 m sheet metal and approximately 1500 holes, it will be completed 12.5 min earlier and the light turn-on times of the laser cutting head and resonator will be reduced, increasing the components’ lifetimes.

**Figure 9:**
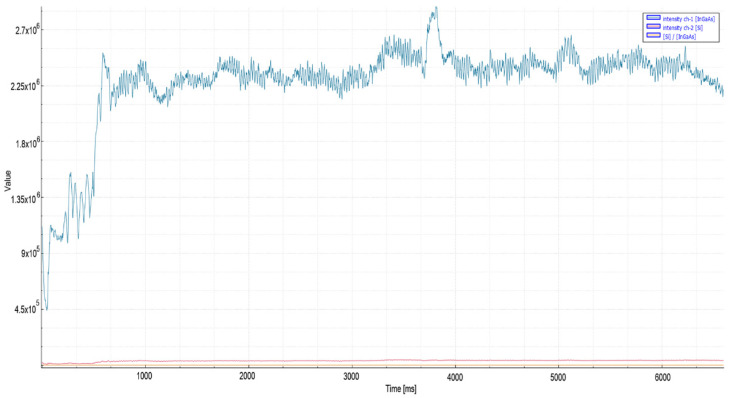
Measurement values recorded during oxygen cutting of 15 mm mild steel material.

**Figure 10:**
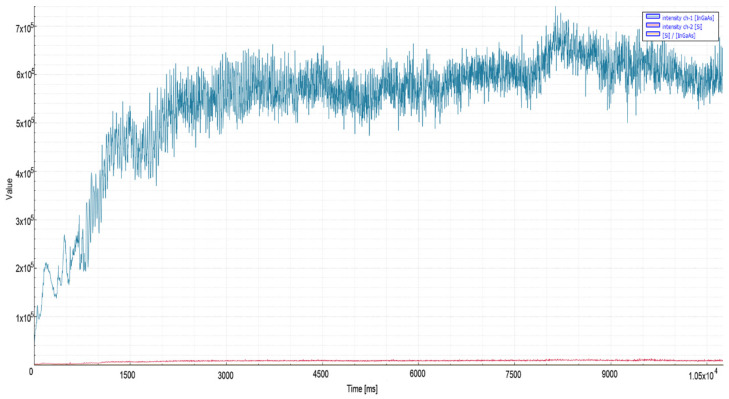
Measurement values recorded during oxygen cutting of 25 mm mild steel material.

As seen in Figure 9, 15 mm mild steel material revealed a higher average signal level during quality cutting (2.3 × 10^6^) than the 25 mm material (6 × 10^5^), as shown in Figure 10. This situation has been associated with the heat conduction phenomenon in the literature [27].

Mild steel material is often cut with oxygen because of the cutting costs and surface quality. However, in order to observe the effects of an increase in laser power and acceleration (Table 6) on the sensor output, 3 and 4 mm mild steel material was cut using 10 kW laser power and nitrogen gas. In both of the materials, a rapid piercing process was applied with the effect of high laser power and nitrogen.

**Figure 11:**
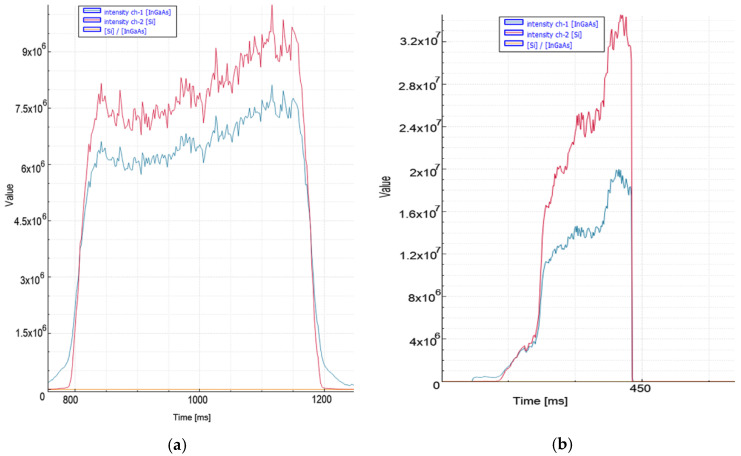
Measurement values recorded during nitrogen cutting of 3 mm mild steel material: (**a**) quality cut with ideal parameters, (**b**) the moment of deterioration of cut and quality.

According to the data obtained, while the signal levels are normal (7.5 × 10^6^) at the time of cutting, there is an increase (1.03 × 10^7^) in the readings with the effect of acceleration. Figure 11 shows the signal characteristic obtained from the photodiodes when the cut quality deteriorates. In the peak levels of the signal, 3.5 times increases are noticeable. Considering the slope of the increase in signal, 1.6 × 10^7^ was chosen for the threshold level, and a 15 ms decision time was considered sufficient.

**Figure 12:**
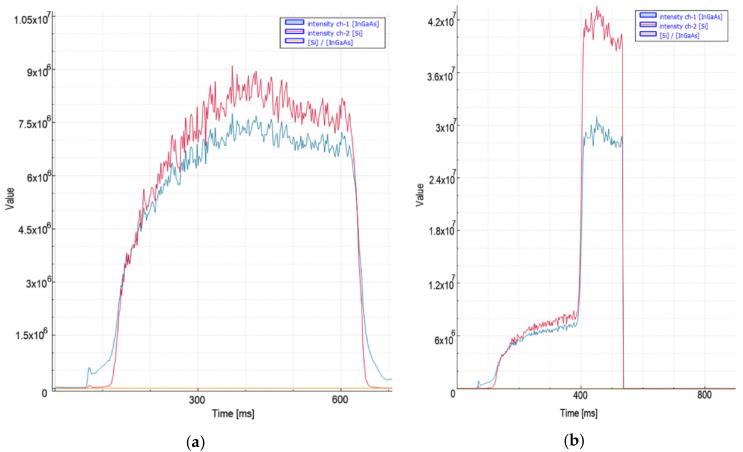
Measurement values recorded during nitrogen cutting of 4 mm mild steel material: (**a**) quality cut with ideal parameters, (**b**) the moment of deterioration of cut and quality.

In the 10 kW nitrogen cuts (Figure 12) of 4 mm mild steel material, a peak level of 9 × 10^6^ was recorded when the process speed was the highest. In the second experiment where the cut quality deteriorated, a 4.2 × 10^7^ level was observed at the output of the Si photodiode in the VIS region. According to the data received, it was deemed appropriate to choose a threshold level of 1.3 × 10^7^ and a decision time of 15 ms. A single step was applied for the nitrogen piercing process in 3 and 4 mm mild steel material. The piercing process, which takes 0.6 s with ideal parameters, is completed in an average of 0.3 s with the applied method.

## 5. Conclusions

In the presence of many factors affecting the processing of metal plates in high-power fiber laser applications, the same main parameters cannot be used to process different materials. The events in the process develop so fast that the human eye cannot perceive them. For this reason, the existence of a system that can monitor the process has recently been an important research topic in the literature. In this article, an improved system is presented which can perform real-time monitoring based on InGaAs (1000–1800 nm) and Si (400–1100 nm) photodiodes and analyze the situation with smart algorithms by integration with these sensors.

In order to understand how the material type, thickness, and applied laser power affect the properties of our system, measurements were made with three different high-power laser power units, dozens of different material types, and various tests. Each material was cut both in quality and in poor quality; only quality piercing parameters were used, as the aim during the piercing process was rapid detection. By choosing the most commonly used material types in the industry, it was revealed that measurements made on the piercing and cutting of different materials and thicknesses, and the piercing and cutting threshold values and the decision time, vary depending on the material properties.

It was observed that the signals observed in the IR and VIS spectra are stable for cases with good cut quality. Average values tended to increase as cut quality deteriorated. It is graphically represented that the poor shear response observed in the stainless steel and aluminum cutting processes is much greater than that observed for oxygen-cut mild steel. However, the most important effect in the nitrogenous section of mild steel is that the signal increase in the VIS spectrum is much more pronounced in the weaker section than in the IR spectrum. This behavior was not observed in other material types.

During the cutting tests, the positions of the axes were also recorded in the machine. In this way, it was possible to match the regular quality deteriorations in the cut workpiece corresponding to the peaks in the monitored signal. In addition, the variability in cut quality, due to sudden turns in the workpiece geometry, was also associated with this. Thus, it was demonstrated that the proposed photodiode system can be used for cutting parameter adjustments in a new material type.

The real-time monitoring system proposed in the article prevents material deterioration that may occur during piercing and cutting by directly interfering with the process in all tests. The tests demonstrated the reliability, stability, and potential of the photodiode-based tracking system. While the piercing detection system increases efficiency and productivity by more than 25% depending on the part complexity, size, and material, the bad cut detection method has become a quality control factor that can ensure the continuity of the process with unmanned automatic intervention without disturbing the workpiece.

## Figures and Tables

**Table 1:** Optical characteristics of laser resonator.

Characteristics	Min.	Typ.	Max.	Unit
Operation Mode	CW/Modulated			
Polarization	Random			
Output Power Tuning Range		10	105	%
Emission Wavelength	1068		1080	nm
Emission Linewidth		3	6	nm
Beam Parameter Product		3.30	4.0	
Switching On/Off Time		50	100	µs
Output Power Modulation Rate			5	kHz
Output Power Instability		±1	±2	%

**Table 2:** Optical output of laser resonator.

Characteristics	Min.	Typ.	Max.	Unit
Delivery Fiber Connector	HLC-8, QBH-compatible LCA, QD-compatible			
Beam Parameter Product (86%)		2.0	2.2	mm × rad
Delivery Fiber Length	10	20	30	m
Delivery Fiber Bending Radius -unstressed -stressed	100 200			mm

**Table 3:** Optical characteristics of laser resonator.

Specifications	Value	Unit
Laser Wavelength	1030–1090	nm
Laser Power	Max. 12,000	W
Collimation Focal Length	100	%
Focusing Focal Length	125, 150, 175, 200	nm
External Acceleration	45	nm
Cutting Gas Pressure	Max. 25	
Auxiliary Gas Pressure	Max. 5	µs
Cooling Gas Pressure	Max. 5	kHz
Operating Voltage	Max. 24, 10% and 4	%

**Table 4:** Example piercing and cutting parameter set used in 6 kW tests.

Parameter Set	8 mm SS	8 mm AL	20 mm MS
Nozzle Distance	7–2 mm	8–1.2 mm	15–2.6 mm
Gas Type/Pressure	Nitrogen/15 Bar	Nitrogen/10 Bar	Oxygen/0.8–1.2 Bar
Focus Type/Distance	F 150/−4.8 mm	F 150/−3.2 mm	F150/4 mm
Laser Power	6000 W	6000 W	6000 W
Duty (%)	60–100	80–100	40–100
Frequency	5 kHz	5 kHz	50 Hz
Cutting Speed	2800 mm/min	3300 mm/min	750 mm/min

**Table 5:** Example piercing and cutting parameter set used in 8 kW tests.

Parameter Set	15 mm MS	25 mm MS
Nozzle Distance	12–2 mm	14–2.5 mm
Gas Type/Pressure	Oxygen/0.5 Bar	Oxygen/0.5–0.8 Bar
Focus Type/Distance	F 200/9 mm	F 150/3 mm
Laser Power	8000 W	8000 W
Duty (%)	30–100	40–100
Frequency	50 Hz	50 Hz
Cutting Speed	1700 mm/min	650 mm/min

**Table 6:** Example piercing and cutting parameter set used in 10 kW tests.

Parameter Set	3 mm MS	4 mm MS
Nozzle Distance	8–1 mm	9–1.2 mm
Gas Type/Pressure	Nitrogen/13 Bar	Nitrogen/13 Bar
Focus Type/Distance	F 150/0.5 mm	F 150/0.2 mm
Laser Power	10,000 W	10,000 W
Duty (%)	70–100	100
Frequency	5 kHz	5 kHz
Cutting Speed	29,000 mm/min	21,300 mm/min

**Figure A1:**
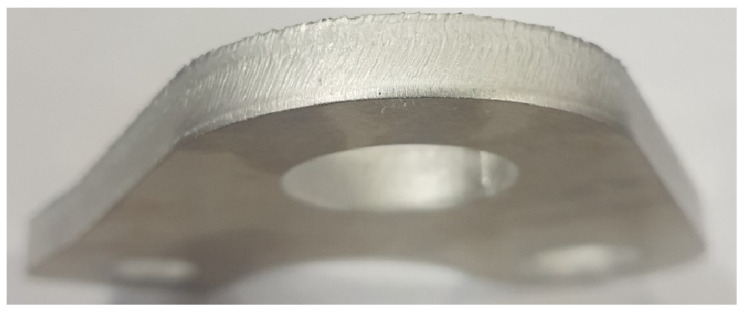
Edge surface quality of the cut 8 mm aluminum workpiece.

**Figure A2:**
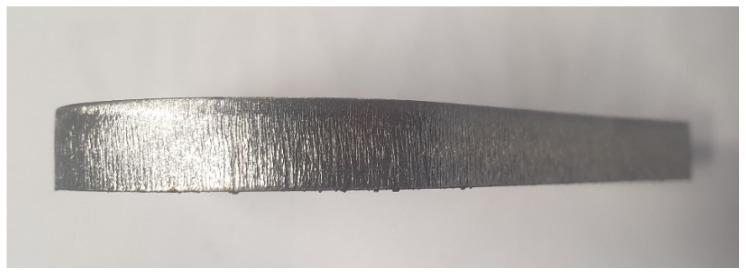
Edge surface quality of the cut 8 mm stainless steel workpiece.

**Figure A3:**
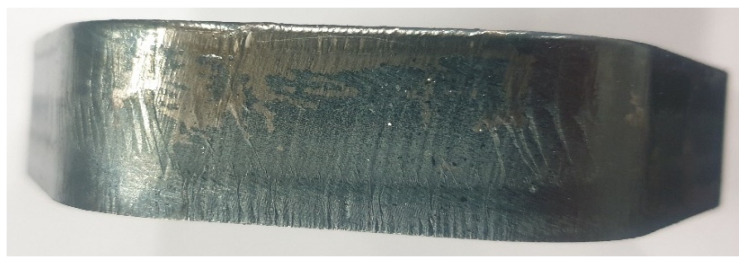
Edge surface quality of the cut 20 mm mild steel workpiece.

**Figure A4:**
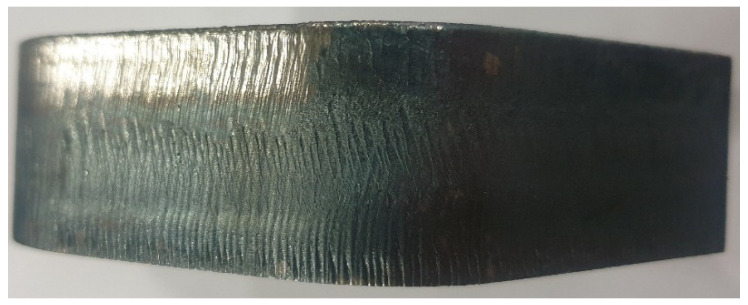
Edge surface quality of the cut 25 mm mild steel workpiece.

**Figure A5:**
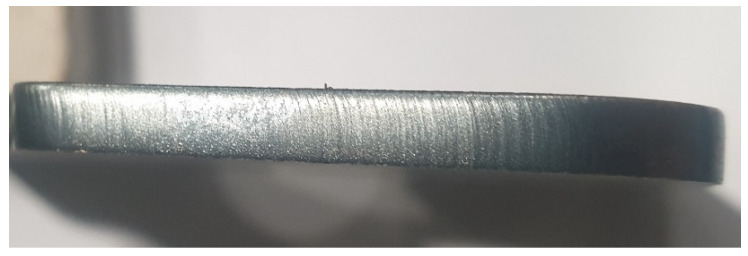
Edge surface quality of 4 mm mild steel workpiece cut with Nitrogen.

**Figure A6:**
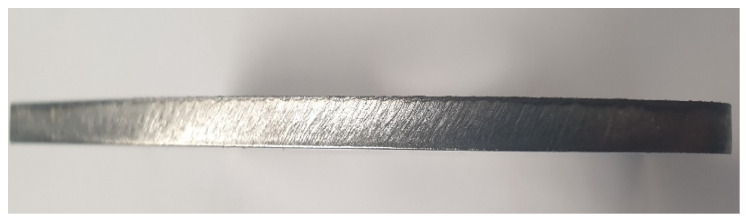
Edge surface quality of 3 mm mild steel workpiece cut with Nitrogen.

## Data Availability

Not applicable.
